# Report of Case of Cancrum Oris

**Published:** 1891-04

**Authors:** W. P. McDonald

**Affiliations:** Hill City, Tenn.


### THE DENTAL REGISTER.

VOL. XLV.] APRIL, 1891. [No. 4.

### COMMUNICATIONS.

### Report of Case of Cancrum Oris.

W. P. MCDONALD, M.D., HILL CITY, TENN.

Read before Tri-State Medical Association, Oct. 14th, 1890, Chattanooga, Tenn.

Ellen Maverty, white, aged 4 years, residence Hill City, Tenn.

Was called to see her first on Aug. 5th, found her with some fever, tongue coated brown, with red edges, surface more or less furrowed or full of cracks in the brown coat; her general appearance indicating a very low state of health. Her bowels were inclined to be too loose and abdomen somewhat distended, tympanitic.

With these symptoms, I at once pronounced it a case of the so-called typho-malarial fever, something not at all rare in the southern states.

I began treatment by giving her a mercurial purge followed by large doses of quinine and sub-nitrate of bismuth. Continued this treatment for two days, then ordered her to have ten drops of the syrup of the iodide of iron after meals, also quinine in small tonic doses.

Kept up this treatment for ten days and found her very much improved, having no fever, regaining her color and with it strength; appetite very good and resting very nicely all the time.

On Aug. 19th, just 14 days after seeing child first time, I was called to see the mother, whom I found with nearly the same symptoms as the child first presented. During my absence from the city another physician had treated an older daughter, whom I suppose had been troubled with the same disease, typho-malarial fever. At that time, the little child, the subject of this report, was still improving, except complaining of sore mouth. On examination I found several small ulcers on the right side of mouth, with a general inflammatory condition of the gums and whole mucous lining of that side of the buccal cavity, with some bleeding from around the teeth.

The trouble at first seemed to yield to a wash of chlorate of potassium and creosote, but on Aug. 23rd, or the 17th day of illness, the inflammation increased rapidly, the whole cheek and side of face appearing very much swollen and feverish; and the inside (as best could be seen,) was fast becoming dark and gangrenous. On the next evening, Aug. 24th, a small dark spot the size of a penny, made its appearance externally, just at the right wing of the nose. This rapidly enlarged, involving the wing of nose and in proportion the tissues on either side of the central spot, and on Aug. 25th it had completely involved the right side of the nose up to the inner canthus of the eye, also the upper lip to the median line, and had spread rapidly on the cheek, reaching a point where the zigomatic muscles cross the superficial portion of the masseter. The teeth on this side became loose and dropped out, both above and below, indicating deep seated trouble, possibly necrosis of the maxillary bones. Just at this stage, the evening of Aug. 25th, the rapid spreading seemed to cease and the whole gangrenous mass commenced to loosen around the edges, still held firmly in place by the blood vessels; as we are taught that, they are lost to give way to decay.

The child's breath was very offensive from the beginning of the mouth trouble, and the odor increased as the disease advanced until the stench was more offensive than a dissecting room well stocked with cadavers.

Up to this time the child had been taking boiled milk, beef tea, etc., in sufficient quantity to support her, but here she refused everything and vomited continually from the watery discharges from the gangrenous mass passing into her stomach. She gradually grew weaker until Sept. 4th when death came to her relief, it being just thirty days from date of first illness, and sixteen days from the beginning of the mouth trouble.

Upon careful investigation I found a strumous diathesis existing in the family, but could get no history of any tuberculosis. The family were in very poor circumstances, and had lived all their lives in small, poorly ventilated houses, and had taken just such food as could be had. Just here let me say that as *gout* is a disease among aristocrats and caused from high living, so is *cancrum oris* a disease among the poverty-stricken and caused *principally* by poor living, so to find a case of either of these diseases—gout or cancrum oris—outside of the spheres of life before mentioned, is the exception and not the rule.

Now, from good authority we have it that cancrum oris occurs more frequently as the sequel of other diseases than *per se.*

The statistics of Pilliet and Barthez show that out of 98 cases of this trouble 41 of them followed measels; 5 scarlet fever; 6 whooping cough; 9 intermittent fever; 9 typhoid fever and 7 mercurial salivation.

Now, to draw a conclusion in this case from these statistics, and by watching the case closely from the first—Did this occur as the *sequel* of the typho-malarial fever which I diagnosed, remembering the poor state of health the child was in to begin with? Or was it the result of the *three grains* of calomel given at first and followed in *two days* by the syr. of the iodide of iron producing mercurial salivation.

